# Serum MicroRNA-21 as a Diagnostic Marker for Lung Carcinoma: A Systematic Review and Meta-Analysis

**DOI:** 10.1371/journal.pone.0097460

**Published:** 2014-05-27

**Authors:** Xiaorong Yang, Yanan Guo, Yane Du, Jinmei Yang, Shichao Li, Shengnan Liu, Ke Li, Dechun Zhang

**Affiliations:** Department of Pathogenic Biology, Chong Qing Medical University, Chongqing, People's Republic of China; University of Verona, Italy

## Abstract

**Objectives:**

MicroRNA-21 in serum is a promising marker for the diagnosis of lung carcinoma. A meta-analysis was performed to assess the diagnostic accuracy and clinical value of serum microRNA-21 in patients with lung carcinoma.

**Methods:**

PubMed, EMBASE, Web of Knowledge (ISI), the Cochrane Library, Scopus, BioMed Central, Science Direct, China National Knowledge Infrastructure (CNKI), Wan Fang data and Technology of Chongqing (VIP) databases were searched to identify studies in English and Chinese that assessed the diagnostic value of serum miR-21 for lung carcinoma, from inception to 9 April 2014. Two independent investigators identified and extracted the study characteristics from all articles according to defined inclusion and exclusion criteria. Quality assessment of diagnostic accuracy studies (QUADAS) was used to score the quality of the eligible studies. Stata12 and Meta-DiSc software were used to test the heterogeneity and to perform the meta-analysis.

**Results:**

Our search returned 1008 articles, of which seven fulfilled the inclusion criteria, accounting for 500 patients and 386 controls. Using random-effect model analysis, the summary assessments revealed that the mean sensitivity was 0.71% (95%CI: 57–82%) and specificity was 0.84% (95%CI: 76–89%). The area under the receiver operating characteristic curve was 0.86 (95%CI: 0.83–0.89). In addition, heterogeneity was clearly apparent but was not caused by the threshold effect, as shown by Meta-DiSc analysis.

**Conclusion:**

The current evidence suggests that serum miR-21 can be rapidly measured in lung carcinoma patients and has potential diagnostic value with moderate sensitivity and specificity. Further prospective studies to assess the early stage diagnostic value are needed in the future.

## Introduction

Lung cancer is one of the most common malignancies worldwide. The American Cancer Society reported that there were 228,190 new lung and bronchus cancer cases and 159,480 new deaths in 2013 [1]. Moreover, lung cancer has become the most common cancer in China. New statistical data, investigated by First Sino-US Tobacco Control Forum for Cancer institutions on 17 March 2013, showed that lung cancer mortality has increased by nearly 10-fold during the past 40 years, and that is has become the top cause of cancer-associated deaths, with a rate of 45.57 cases per 100,000 people. Especially in male patients, the mortality rate is as high as 61.00 cases per 100,000 individuals. However, the available treatment options remain insufficient. Comprehensive treatment based on surgery can significantly prolong survival in patients with stage I non-small cell lung cancer (NSCLC), and 5-year survival rates have improved to 45–65% [2]. Unfortunately, there is no ideal detection method for lung cancer diagnosis, especially for early stage lung patients when there are no obvious symptoms. At present, clinicians need to combine a variety of test results to determine whether a patient has lung cancer, and to distinguish the grade and/or stage of the disease. These methods currently include clinical manifestation, physical examination, imaging, endoscopy, among others. However, there is no method available that detects the disease with both high sensitivity and specificity. Thus, novel diagnostic technologies are urgently required. Tumor markers for lung cancer in the blood have become a major focus. Some biomarkers, as carcinoembryonic antigen (CEA), neuron specific enolase (NSE), cytokeratin fragment (CYFRA21-1), and tissue polypeptide specific antigen (TPS), have been used in the clinic, but also lack sufficient sensitivity and specificity.

MicroRNAs (mirRNAs, miRNAs) are a large family of non-coding RNA of approximately 19–25 nucleotides. MiRNA genes first synthesize the primary transcripts (pri-miRNA) in the nucleus [3], then form precursor miRNA (pre-miRNA) after cleavage by Drosha, and are transported to the cytoplasm, where mature miRNA is produced following cleavage by Dicer [4], [5]. MicroRNAs play a pivotal regulatory role in gene expression after mature miRNA partially from complementary pairing with 3′ untranslated region (3′UTR) of the the target mRNA [6]. MicroRNAs have been closely associated with the development of a variety of tumors, such as lung cancer [7], gastric cancer [8], breast cancer [9], nasopharyngeal carcinoma [10], neuroblastoma [11], thyroid cancer [12], leukemia, liver cancer, and colorectal cancer [13], [14]. Furthermore, tumor-associated microRNAs remain stable in serum [15], [16] and have been shown to be advantageous in the diagnosis of tumor diseases; in particular, they have drawn much attention in the field of lung cancer because of their differential expression characteristics. Quantitative real-time reverse transcription polymerase chain reaction (RT-qPCR) exhibits high sensitivity and throughput, good reproducibility and specificity of nucleic acid molecule detection, and can achieve rapid results. Therefore, it has become the gold standard for quantitative detection of serum miRNA in basic and clinical research [17], [18].

MicroRNA-21 (miR-21) is a frequently studied microRNA in lung cancer, and is considered to have an important role in the progression of lung cancer. Wang et al. [19] reported that miRNA-21 in NSCLC patient serum was significantly higher than that in the healthy control group, and its high expression level was correlated with lymph node metastasis and lymph node staging. These data have also been reproduced in other studies [20], [21], which showed that miRNA-21can be used as a diagnosis biomarker for early stage NSCLC. Furthermore, Le et al. [22] found that postoperative serum miR-21 levels decreased significantly compared with that in preoperative serum concomitant with shorter survival in patients with lung carcinoma, which suggested that miR-21can be used to predict the risk of lung cancer recurrence. Gao et al. [23] investigated miR-21 expression in patients with lung squamous cell carcinoma, and found high expression of miR-21 also indicated a poor prognosis.

To date, many researchers have published their data for the diagnostic value of miR-21 in lung cancer, and have raised concerns about the efficiency of miR-21 as a biomarker. In this study, we performed a meta-analysis of these published studies to estimate the diagnostic value of miR-21 in lung cancer.

## Methods

### Search strategy and selection criteria

We systematically searched PubMed, EMBASE, Web of Knowledge (ISI), the Cochrane Library, Scopus, BioMed Central, Science Direct, China National Knowledge Infrastructure (CNKI), Wan Fang data and Technology of Chongqing (VIP) database for studies that assessed the diagnostic value of miR-21 in lung cancer. Existing systematic reviews, meta-analysis and bibliography of the reports were also checked for potentially relevant additional studies. The studies were restricted to those in English and Chinese only.

The following selection criteria were used to search articles and abstracts: (‘miR-21’ or ‘microrna-21’ or ‘Hsa-mir-21’) and ‘lung’ and (‘cancer’ or ‘carcinoma’ or ‘tumor’ or ‘neoplasm’ or ‘cancers’). Databases were searched between their inception and 9 April 2014.

### Data extraction and quality assessment

Inclusion criteria were as follows: 1) Measurement of serum of miR-21 in lung cancer. 2) The appropriate gold standard method confirmed the case group and patients with lung cancer and that the control group was disease-free. 3) Sensitivity and specificity were reported to provide sufficient information to construct 2×2 contingency tables. The table includes false and true positive and negative information. Animal experiments, reviews, meta-analysis and conferences were excluded, as previously described [24].

Two investigators independently extracted data in the study to obtain information, which included study details such as first author, year of publication, disease type, methods and cut-off value, and data for a 2×2 table and study design. If further information was needed, the corresponding authors were contacted.

The Quality Assessment of Diagnostic Accuracy Studies (QUADAS) was used to assess each study for the quality of the information reported [25].

### Statistical analysis

Stata12 and Meta-DiSc software were used to test the heterogeneity and to perform the meta-analysis [26]. We tabulated 2×2 contingency tables, which contained information regarding true positives, false negatives, false positives and true negatives, to calculate pool sensitivity, pool specificity and a corresponding CI. The Spearman model was applied to assess heterogeneity caused by different cut-off values. Forest plots, Cochran-Q value and I^2^ test were adopted to estimate heterogeneity caused by others factors. We also introduced funnel plots, Egger's test and Begg's test to investigate publication bias. *P*-values of <0.50 indicated significant heterogeneity [27], [28].

## Results

### Data extraction

**Figure 1:**
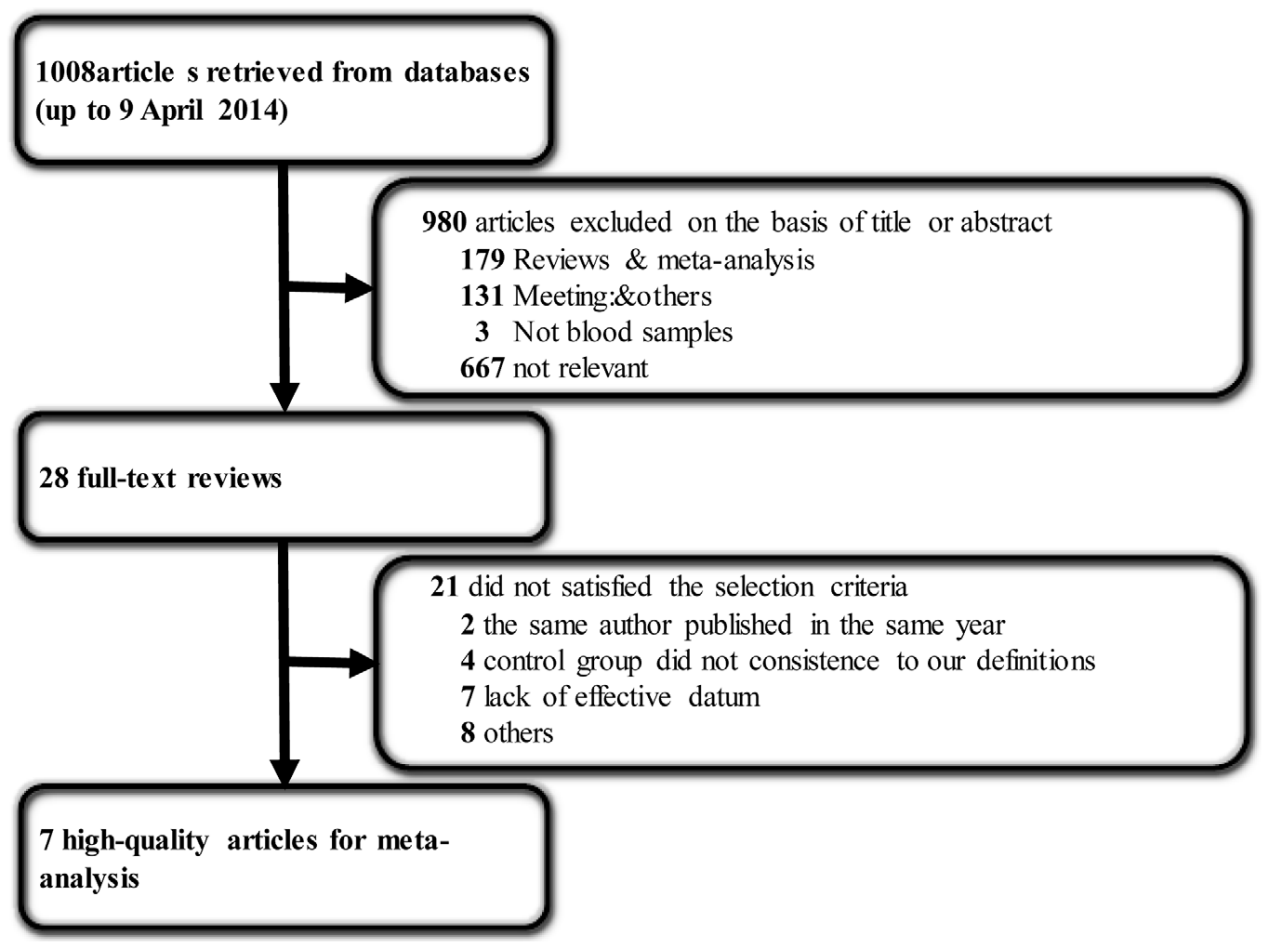
Flow diagram of the selected eligible studies based on the inclusion and exclusion criteria.

A total of 1008 articles were retrieved from the databases. After reviewing the titles and abstracts, 980 articles were excluded, including 179 reviews and meta-analysis, 131 meetings and others, and three no-blood samples. After reviewing the full text according to the selection criteria, 21 articles further excluded, seven of which contained imperfect information needed to meet the requirements of the inclusion criteria. Three articles published were by the same author in 2011 [29]–[31], hence we selected the article that examined the most samples [30]. Finally, seven articles [22], [30], [32]–[36] containing 868 samples remained (Figure 1). Since some did not have cut-off values, we contacted the corresponding author to ask for the information, and all replied.

### Data characteristics and quality assessment

Table 1 shows the eligible characteristics of articles that were published between 2011 and 2013. All 500 lung cancer patients were confirmed by histology, which is the gold standard for lung cancer diagnosis. Lung cancer stages were classified according to the National Comprehensive Cancer Network (NCCN) or the Union for International Cancer Control (UICC) in 2010 and/or American Joint Committee on Cancer (AJCC, 7th edition) in 2009. A total of 368 normal volunteers showed no characteristics of lung cancer during their medical checkup, including 92 healthy smokers and 30 benign pulmonary nodules [33]. RT-qPCR was used to detect miR-21 expression in human peripheral blood. Most data were normalized using the 2-ΔΔCt method, with the exception of that produced by Amal A. et al., which was normalized using the ΔCt method [35]. The cut-off values or expression levels are presented in Table 1.

**Table 1:** Summary of studies using miR-21 as a biomarker of lung carcinoma and study quality assessment.

First author	Year	patients (controls)	Racial	Disease	Cut-off	Normalizers	RNA extraction kits	Measurements	TP	FP	FN	TN	QUADAS scores
Hanbo Le et al. [22]	2012	82(50)	China	lung cancer	1.68 2^−ΔΔCt^	miRNA-16	miRNA extraction kit	TaqMan	38	4	44	46	13
Yanzhao Li et al. [32]	2011	20(10)	China	lung cancer	1.947 ng/ml	miRNA mimics	miRNA extraction kit	SYBR	16	0	4	10	12
Dongfang Tang et al. [33]	2013	96(122)	China	lung cancer	1.31	small nuclear U6B RNA	----	TaqMan	48	34	48	88	13
Bing Wang et al. [34]	2012	31(30)	China	lung cancer	3.63 2^−ΔΔCt^	miRNA-16	TRIzol	SYBR	27	8	4	22	13
Juan Wei et al. [30]	2011	77(36)	China	NSCLC	1.63 2^−ΔΔCt^	Cel-miR-39	miRNA extraction kit	SYBR	47	6	30	30	13
Amal A. et al. [35]	2013	65(37)	Egypt	lung cancer	2.441 ΔCt	miRNA SNORD68	miRNA extraction kit	SYBR	56	5	9	32	13
Hui Zhang et al. [36]	2012	129(83)	China	NSCLC (stageI, II)	1.101 2^−ΔΔCt^	Cel-miR-39	TRIzol	SYBR	100	12	29	71	13

**Note:** NSCLC: non-small cell lung cancer. TP: true positive. FP: false positive. FN: false negative. TN: true negative. QUADAS: The Quality Assessment of Diagnostic Accuracy Studies. ----: not reported in study.

In addition, Han-Bo Le et al. [22], Hui Zhang et al. [36] and Juan Wei et al. [29] compared miR-21 expression in the sera of 237 patients with early stage disease (I and II) against healthy individuals. Dongfang Tang et al. [33] set a training group and validation group independently. Thus, we calculated 62 patients in the training set and 34 malignant tumor patients in the validated set by their respective given information as patients cases of this study, while 60 healthy smokers of the training set, 32 healthy smokers and 30 patients with benign pulmonary nodules of the validated set were calculated as control cases. Strikingly, the training group and validation group had the same cut-off value, but did not have a pool AUC value when combined. Moreover, Han-Bo Le et al. [22] and Hui Zhang et al. [36] investigated the different levels between pre- and post-operative lung cancer patients, totaling 102 cases. QUADAS scores of studies were from 12 to 13, which satisfy the majority of the standards, as no one reported the interval period between the reference standard and index test to ensure that the target conditions did not change during the two tests. Yanzhao Li et al. [32] did not described sufficient details of histology method used to enable its replication.

### Heterogeneity analysis and diagnostic value of miR-21

**Figure 2:**
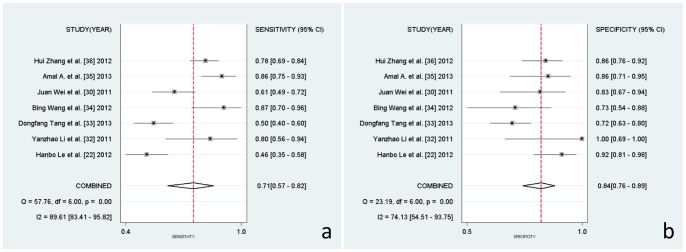
Forest plots of sensitivities and specificities for miR-21 test accuracy in lung carcinoma.

As shown by the forest plot of sensitivity and specificity (Figure. 2) for miR-21 testing in lung carcinoma, the heterogeneity analysis revealed I^2^ values of 90.54 (95%CI 85.04–96.04; *P* = 0.000) for sensitivity and 74.30(95%CI 54.84–93.76; *P* = 0.000) for specificity. This provided evidences of high levels of heterogeneity in the seven studies [27]. However, the Spearman value was found to be 0.14 (*P* = 0.76), It implying that the heterogeneity was not caused by the threshold effect. Meta-regression analysis was used to identify if the heterogeneity was caused by ethnic background and/or the miRNA normalizer and/or different methods of RT-qPCR, which are the most important causes of heterogeneity. As shown in Table 2, the data suggest that the ethnic background of patient samples did not cause heterogeneity, nor did the normalizers, RNA extraction kits, or RT-qPCR measurements.

**Table 2:** RDOR and P-values of covariants in the meta-regression analysis.

	RDOR	p-Value
Racial	26.48	0.30
Normalizers	0.37	0.32
RNA extraction kits	3.10	0.44
Measurements	12.66	0.27

**Note:** RDOR: relative diagnostic odds ratio.

**Figure 3:**
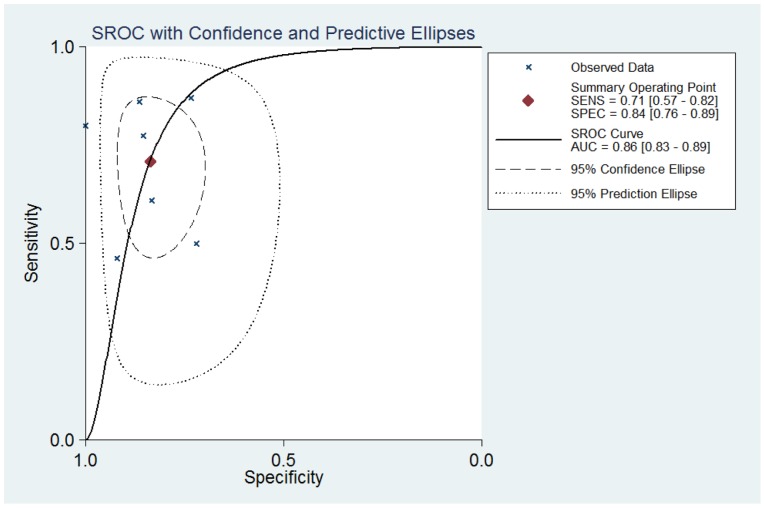
Summary ROC curve of miR-21 diagnostic value in lung carcinoma.

**Figure 4:**
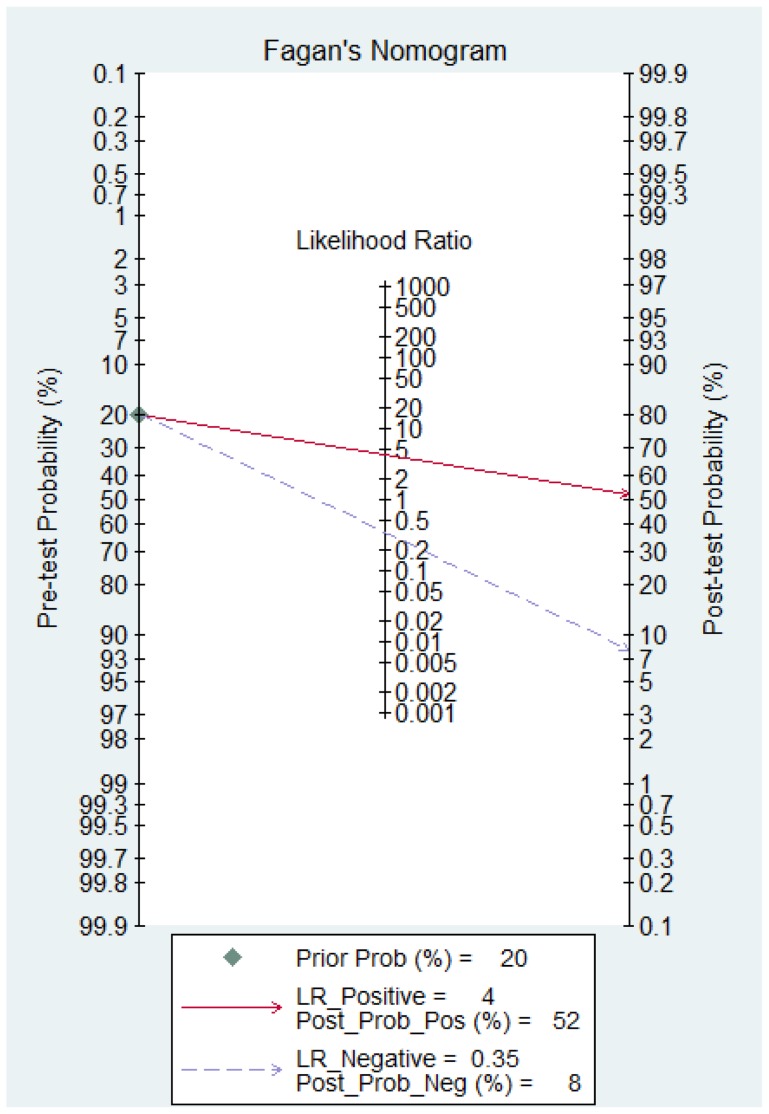
Fagan's Nomogram for assessment of post-test probabilities (PTPs).

**Figure 5:**
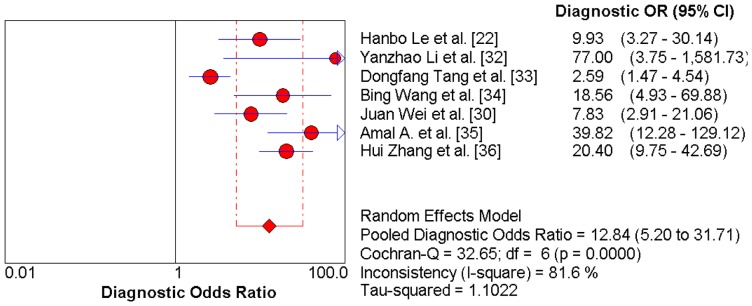
Diagnostic Odds Ratio with Cochran-Q value.

Thus, the random effects model approach was used in this meta-analysis to eliminate heterogeneity [37]. As a result, the summary assessment of miR-21 in the diagnosis of lung carcinoma showed that the pooled sensitivity was 0.71 (95%CI 0.57–0.82) and the pooled specificity was 0.84 (95%CI 0.76–0.89). The area under the summary receiver operating characteristic curve was 0.86 (95%CI 0.83–0.89; Figure. 3). These data indicated that the diagnostic capability of miR-21 in lung cancer is moderate. Likewise, both the likelihood ratios and post-test probabilities were moderate (Figure. 4). The positive likelihood ratio of 4 implies that a person with lung cancer is four-times more likely to have a positive test result than a healthy person. Given a pre-test probability of 20%, the post-test probability of lung cancer for a positive test result is 52%, while a negative test result is 8%. Meanwhile, the diagnostic odds ratio value was 12.58 (95%CI: 5.76–27.48), indicating miR-21 can be used as a good indicator of lung carcinoma diagnosis (Figure. 5).

### Publication bias

Funnel plots were calculated to access the publication bias of the studies (**Fig. S1**). The shape of funnel plots revealed symmetry despite the limited study number. Egger's and Begg's tests were also used to additionally highlight the publication bias by quantitative methods in the meta-analysis. *P* values of the Egger's and Begg's tests were 0.058 and 0.548, respectively, which suggested no publication bias for the meta-analysis in the use of miR-21 in lung carcinoma diagnosis in a limited number of eligible studies.

## Discussion

MiR-21 as a novel biomarker of diagnosis in lung cancer has generated much interest. The differential diagnostic value of miR-21 for lung cancer patients has been observed with inconsistent conclusions. However, no meta-analysis has previously been reported. Following the acquisition of a large sample size and appropriate method to synthesis the individual data, a meta-analysis to assess the role of miR-21 in the diagnosis of lung cancer was performed.

Seven studies were included in this meta-analysis. They satisfied all QUADAS requirements except the fourth, which did not show the interval period between reference standard and index test. Samples of peripheral blood derived during the patients' initial assessments were confirmed as lung cancer before definitive adjuvant therapy and/or surgical intervention. However, it was unknown whether the index test was taken in time. Heterogeneity is another major problem in interpreting the results of a meta-analysis. The threshold effect must be considered as the first factor in testing the accuracy of a study; we used Spearman and Cochran-Q test, and found Spearman value of 0.00 (*P* = 1.00) using Meta-Disc analysis, as there was zero value existing in the FP of Yanzhao Li et al. [32] study. We changed 0 to 1, the minimum value of this research, which corrected the Spearman value to 0.14 (P = 0.76). This implied that the heterogeneity was not caused by the threshold effect. The Cochran-Q value was 32.65 (*P* = 0.00), which is the same result as the Spearman value. One-way sensitivity analysis (data not shown) was implemented to identify the factors that caused heterogeneity, but it failed. Thus, a random-effects model was used to eliminate some heterogeneity. In addition, there are other limitations in this meta-analysis. Some data, such as conference abstracts, non-English and Chinese literature, unpublished data and other inconsistent reports according to our selection criteria were excluded. All may cause publication bias to some degree, although there is no publication bias for the funnel plots and Egger's and Begg's tests. In summary, our data imply a moderate value of miR-21 in lung carcinoma diagnosis [27], [39].

Moreover, the lung cancer analyzed in the meta-analysis was general, and included squamous cell carcinomas (SCC) and adenocarcinomas amongst other types. MiR-21 expression level in plasma varies with the developmental stage of the disease, and there is insufficient data to analyze each stage of each lung cancer by meta-analysis. Han-Bo Le et al. [22] and Hui Zhang et al. [36] both compared the plasma miR-21 expression between pre- and post-operative patients with lung carcinoma, and between early stage lung carcinoma patients (stage I–II) and healthy volunteers. They obtained comparable results that showed the following: 1). the expression level of miR-21 was more significantly decreased in post-operative plasma than in paired pre-operative sample; 2). the expression level of miR-21 was more increased in lung carcinoma plasma samples than healthy volunteers. The sensitivity and specificity of miR-21 in lung carcinoma diagnosis reported by Han-Bo Le et al. [22] were 47.5% and 88%, respectively, while those reported by Hui Zhang et al. [36] were 77.5% and 85.5%, respectively. Interestingly, Juan Wei et al. [29] also completed similar research and reported that miR­21 expression in plasma was higher in stage III–IV patients than in stage I–II patients and higher in SD and PD patients than in PR patients, who, with advanced NSCLC, were treated with platinum chemotherapy. In summary, these results suggest that miR-21 may serve as a potential novel non-invasive biomarker for the diagnosis of lung cancer and in the development of lung cancer treatment. Unfortunately, we fail to obtain a 2×2 table or sensitivity and specificity value from Juan Wei et al. [29], thus there were insufficient articles to perform a meta-analysis. Therefore, additional reports of early stage diagnosis and prognosis are needed.

In conclusion, miR-21 is a promising biomarker for the diagnosis of lung carcinoma. Its sensitivity and specificity are both higher than that of single traditional diagnostic markers, such as CEA, NSE, CYFRA-21 and TPS [38]. It can also be assessed more rapidly and less invasively than other markers that require analysis by histopathology. As no ideal biomarker currently exists for lung carcinoma miR-21 can be used as a preferred diagnostic biomarker.

## Supporting Information

Figure S1 **Funnel plots for the assessment of potential bias in miR-21 assays.**
(TIF)

Checklist S1 **Prisma Checklist.**
(DOC)

## References

[1] Siegel R, Naishadham D, Jemal A (2013) Cancer statistics, 2013. CA Cancer J Clin 63: 11–30. 23335087 10.3322/caac.21166

[2] Ettinger DS, Akerley W, Bepler G, Blum MG, Chang A, et al (2010) Non-small cell lung cancer. J Natl Compr Canc Netw 8: 740–801. 20679538 10.6004/jnccn.2010.0056

[3] Lee Y, Ahn C, Han J, Choi H, Kim J, et al (2003) The nuclear RNase III Drosha initiates microRNA processing. Nature 425: 415–419. 14508493 10.1038/nature01957

[4] Lund E, Guttinger S, Calado A, Dahlberg JE, Kutay U (2004) Nuclear export of microRNA precursors. Science 303: 95–98. 14631048 10.1126/science.1090599

[5] Lee Y, Jeon K, Lee JT, Kim S, Kim VN (2002) MicroRNA maturation: stepwise processing and subcellular localization. EMBO J 21: 4663–4670. 12198168 10.1093/emboj/cdf476 PMC126204

[6] Rana TM (2007) Illuminating the silence: understanding the structure and function of small RNAs. Nat Rev Mol Cell Biol 8: 23–36. 17183358 10.1038/nrm2085

[7] Yu SL, Chen HY, Chang GC, Chen CY, Chen HW, et al (2008) MicroRNA signature predicts survival and relapse in lung cancer. Cancer Cell 13: 48–57. 18167339 10.1016/j.ccr.2007.12.008

[8] Rotkrua P, Shimada S, Mogushi K, Akiyama Y, Tanaka H, et al (2013) Circulating microRNAs as biomarkers for early detection of diffuse-type gastric cancer using a mouse model. British Journal of Cancer 108: 932–940. 23385731 10.1038/bjc.2013.30 PMC3590667

[9] Iorio MV, Ferracin M, Liu CG, Veronese A, Spizzo R, et al (2005) MicroRNA gene expression deregulation in human breast cancer. Cancer Res 65: 7065–7070. 16103053 10.1158/0008-5472.CAN-05-1783

[10] Liu N, Chen NY, Cui RX, Li WF, Li Y, et al (2012) Prognostic value of a microRNA signature in nasopharyngeal carcinoma: a microRNA expression analysis. Lancet Oncol 13: 633–641. 22560814 10.1016/S1470-2045(12)70102-X

[11] Chen Y, Stallings RL (2007) Differential patterns of microRNA expression in neuroblastoma are correlated with prognosis, differentiation, and apoptosis. Cancer Res 67: 976–983. 17283129 10.1158/0008-5472.CAN-06-3667

[12] Jazdzewski K, Murray EL, Franssila K, Jarzab B, Schoenberg DR, et al (2008) Common SNP in pre-miR-146a decreases mature miR expression and predisposes to papillary thyroid carcinoma. Proc Natl Acad Sci U S A 105: 7269–7274. 18474871 10.1073/pnas.0802682105 PMC2438239

[13] Calin GA, Sevignani C, Dumitru CD, Hyslop T, Noch E, et al (2004) Human microRNA genes are frequently located at fragile sites and genomic regions involved in cancers. Proceedings of the National Academy of Sciences of the United States of America 101: 2999–3004. 14973191 10.1073/pnas.0307323101 PMC365734

[14] Lu J, Getz G, Miska EA, Alvarez-Saavedra E, Lamb J, et al (2005) MicroRNA expression profiles classify human cancers. Nature 435: 834–838. 15944708 10.1038/nature03702

[15] Chen X, Ba Y, Ma L, Cai X, Yin Y, et al (2008) Characterization of microRNAs in serum: a novel class of biomarkers for diagnosis of cancer and other diseases. Cell Res 18: 997–1006. 18766170 10.1038/cr.2008.282

[16] Filipowicz W, Bhattacharyya SN, Sonenberg N (2008) Mechanisms of post-transcriptional regulation by microRNAs: are the answers in sight? Nature Reviews Genetics 9: 102–114. 10.1038/nrg2290 18197166

[17] Liu CG, Calin GA, Meloon B, Gamliel N, Sevignani C, et al (2004) An oligonucleotide microchip for genome-wide microRNA profiling in human and mouse tissues. Proc Natl Acad Sci U S A 101: 9740–9744. 15210942 10.1073/pnas.0403293101 PMC470744

[18] Jamnikar Ciglenecki U, Grom J, Toplak I, Jemersic L, Barlic-Maganja D (2008) Real-time RT-PCR assay for rapid and specific detection of classical swine fever virus: comparison of SYBR Green and TaqMan MGB detection methods using novel MGB probes. J Virol Methods 147: 257–264. 18001851 10.1016/j.jviromet.2007.09.017

[19] Wang ZX, Bian HB, Wang JR, Cheng ZX, Wang KM, et al (2011) Prognostic significance of serum miRNA-21 expression in human non-small cell lung cancer. J Surg Oncol 104: 847–851. 21721011 10.1002/jso.22008

[20] Wei J, Gao W, Zhu C, Liu Y, Mei Z, et al (2011) Identification of plasma microRNA-21 as a biomarker for early detection and chemosensitivity of non-small cell lung cancer. Chin J Cancer 30: 407. 21627863 10.5732/cjc.010.10522 PMC4013415

[21] Liu XG, Zhu WY, Huang YY, Ma LN, Zhou SQ, et al (2012) High expression of serum miR-21 and tumor miR-200c associated with poor prognosis in patients with lung cancer. Med Oncol 29: 618–626. 21516486 10.1007/s12032-011-9923-y

[22] Le HB, Zhu WY, Chen DD, He JY, Huang YY, et al (2012) Evaluation of dynamic change of serum miR-21 and miR-24 in pre- and post-operative lung carcinoma patients. Med Oncol 29: 3190–3197. 22782668 10.1007/s12032-012-0303-z

[23] Gao W, Shen H, Liu L, Xu J, Shu Y (2011) MiR-21 overexpression in human primary squamous cell lung carcinoma is associated with poor patient prognosis. J Cancer Res Clin Oncol 137: 557–566. 20508945 10.1007/s00432-010-0918-4 PMC11828261

[24] Yang X, Huang H, Zeng Z, Zhao L, Hu P, et al (2013) Diagnostic value of bladder tumor fibronectin in patients with bladder tumor: a systematic review with meta-analysis. Clin Biochem 46: 1377–1382. 23735602 10.1016/j.clinbiochem.2013.05.064

[25] Whiting P, Rutjes AW, Reitsma JB, Bossuyt PM, Kleijnen J (2003) The development of QUADAS: a tool for the quality assessment of studies of diagnostic accuracy included in systematic reviews. BMC Med Res Methodol 3: 25. 14606960 10.1186/1471-2288-3-25 PMC305345

[26] Zamora J, Abraira V, Muriel A, Khan K, Coomarasamy A (2006) Meta-DiSc: a software for meta-analysis of test accuracy data. BMC Med Res Methodol 6: 31. 16836745 10.1186/1471-2288-6-31 PMC1552081

[27] Higgins JP, Thompson SG, Deeks JJ, Altman DG (2003) Measuring inconsistency in meta-analyses. BMJ 327: 557–560. 12958120 10.1136/bmj.327.7414.557 PMC192859

[28] Dinnes J, Deeks J, Kirby J, Roderick P (2005) A methodological review of how heterogeneity has been examined in systematic reviews of diagnostic test accuracy. Health Technology Assessment 9: 1–113. 10.3310/hta9120 15774235

[29] Wei J, Gao W, Zhu CJ, Liu YQ, Mei Z, et al (2011) Identification of plasma microRNA-21 as a biomarker for early detection and chemosensitivity of non-small cell lung cancer. Chin J Cancer 30: 407–414. 21627863 10.5732/cjc.010.10522 PMC4013415

[30] Wei J, Liu LK, Gao W, Zhu CJ, Liu YQ, et al (2011) Reduction of Plasma MicroRNA-21 is Associated with Chemotherapeutic Response in Patients with Non-small Cell Lung Cancer. Chin J Cancer Res 23: 123–128. 23483517 10.1007/s11670-011-0123-2 PMC3587548

[31] Wei j (2011) Identification of Plasma miRNA-21 as a Biomarker for Early Detection and Chemosensitivity of non-small Cell Lung Caner [Master]: Nan Jing Medical University. 10.5732/cjc.010.10522 PMC4013415 21627863

[32] Li Y, Li W, Ouyang Q, Hu S, Tang J (2011) Detection of lung cancer with blood microRNA-21 expression levels in Chinese population. Oncol Lett 2: 991–994. 22866162 10.3892/ol.2011.351 PMC3408024

[33] Tang D, Shen Y, Wang M, Yang R, Wang Z, et al (2013) Identification of plasma microRNAs as novel noninvasive biomarkers for early detection of lung cancer. Eur J Cancer Prev 22: 540–548. 23462458 10.1097/CEJ.0b013e32835f3be9

[34] Wang B, Zhang Q (2012) The expression and clinical significance of circulating microRNA-21 in serum of five solid tumors. J Cancer Res Clin Oncol 138: 1659–1666. 22638884 10.1007/s00432-012-1244-9 PMC11824721

[35] Abd-El-Fattah AA, Sadik NAH, Shaker OG, Aboulftouh ML (2013) Differential MicroRNAs Expression in Serum of Patients with Lung Cancer, Pulmonary Tuberculosis, and Pneumonia. Cell Biochemistry and Biophysics: 1–10. 10.1007/s12013-013-9575-y 23559272

[36] Zhang H (2012) The study on plasma miR-145, miR-20a, miR-21 and miR-223 as non-invasive biomarkers for early-stage non-small cell lung cancer [Master]: Soochow University.

[37] DerSimonian R, Kacker R (2007) Random-effects model for meta-analysis of clinical trials: an update. Contemporary clinical trials 28: 105–114. 16807131 10.1016/j.cct.2006.04.004

[38] Guo SJ, Li DD, Wen FQ, et al (2013) Diagnostic accuracy of tissue polypeptide specific antigen for small cell lung cancer: a meta-analysis. Med J West Chian 25: 8–11.

[39] Jones CM, Athanasiou T (2005) Summary receiver operating characteristic curve analysis techniques in the evaluation of diagnostic tests. Ann Thorac Surg 79: 16–20. 15620907 10.1016/j.athoracsur.2004.09.040

