# Supplementary figures and images for: Serum MicroRNA-21 as a Diagnostic Marker for Lung Carcinoma: A Systematic Review and Meta-Analysis
Source: PLoS One. 2014 May 27;9(5):e97460. doi: 10.1371/journal.pone.0097460 (PMC4035254; doi:10.1371/journal.pone.0097460)

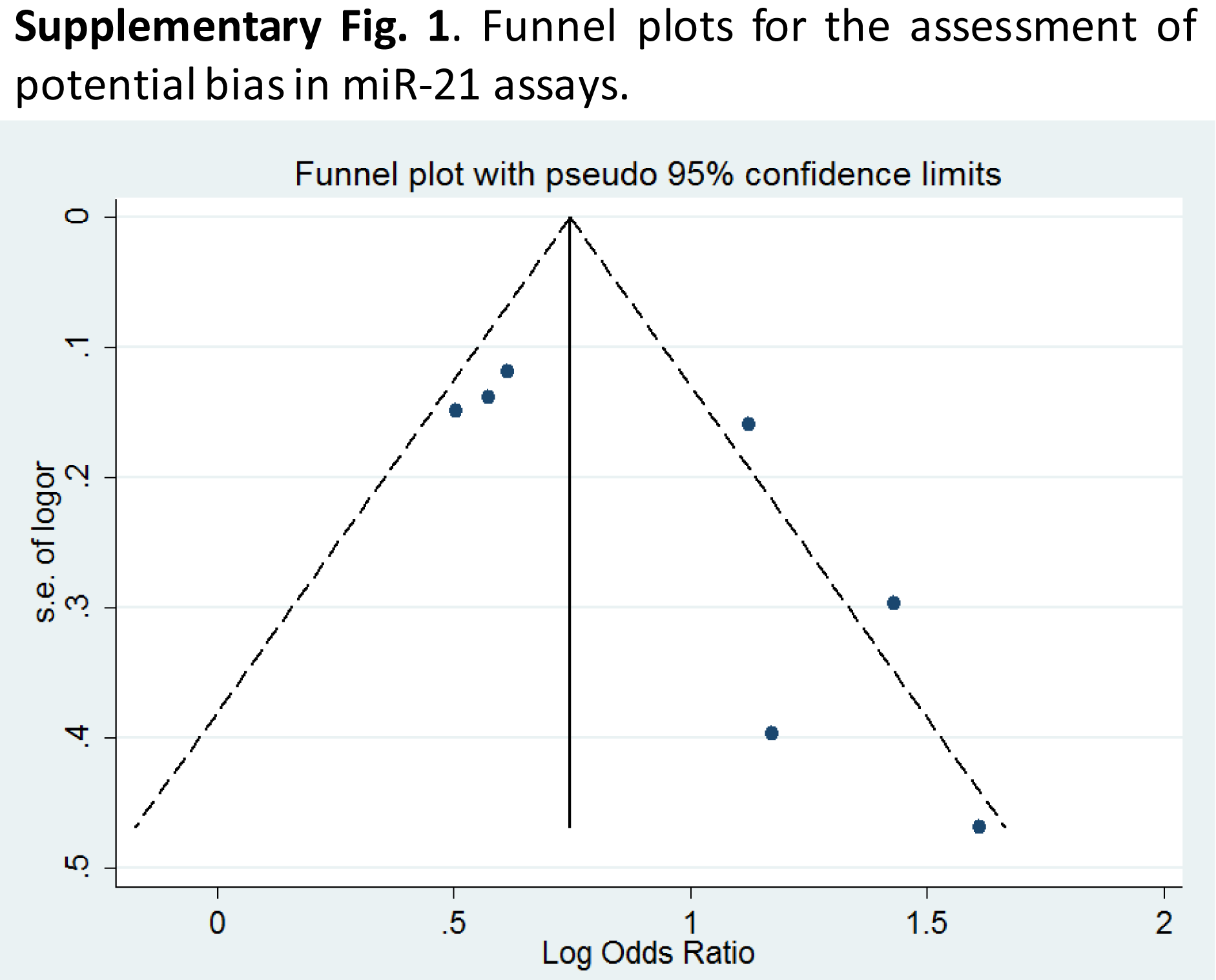

Supplement: Figure S1 — Funnel plots for the assessment of potential bias in miR-21 assays. (TIF) [file pone.0097460.s001.tif]
