# Supplementary material for: Serum MicroRNA-21 as a Diagnostic Marker for Lung Carcinoma: A Systematic Review and Meta-Analysis
Source: PLoS One. 2014 May 27;9(5):e97460. doi: 10.1371/journal.pone.0097460 (PMC4035254; doi:10.1371/journal.pone.0097460)
Supplement: Checklist S1 — Prisma Checklist. (DOC) [file pone.0097460.s002.doc]

| **Section/topic** | **#** | **Checklist item** | **Reported on page #** |
| --- | --- | --- | --- |
| **TITLE** | | |  |
| Title | 1 | **Serum microRNA-21 as a diagnostic marker for lung carcinoma: a systematic review and meta-analysis** | Title |
| **ABSTRACT** | | |  |
| Structured summary | 2 | **Introduction:** Accumulating evidence indicates that microRNA-21(mir-21) is present in high concentrations in plasma of lung cancer patients, suggesting it may be a useful novel molecular diagnostic biomarker of lung cancer. We aimed to assess the potential diagnostic value of mir-21 for lung cancer.  **Methods:** search papers, extract data, Data analysis.  **Results:** the pooled sensitivity was 0.71% and specificity was 0.84%. In addition, the area under the summary ROC curve was 0.86, and DOR was 12.58.  **Conclusions:** The current evidence suggests that mir-21 has potential diagnostic value with a moderate sensitivity and specificity for GC. More prospective studies about the diagnostic value of mir-21 for lung cancer are needed in future. | Abstract |
| **INTRODUCTION** | | |  |
| Rationale | 3 | Numerous groups have now published their experience with the mir-21 test in the diagnosis of lung cancer, and varying results raise concerns about the mir-21 as a biomarker in the early detection of lung cancer. | Introduction |
| Objectives | 4 | So own aim is to explore the potential value of mir-21 in the diagnosis of lung cancer by this meta-analysis, which, to our knowledge, has not previously been performed. | Introduction |
| **METHODS** | | |  |
| Protocol and registration | 5 | There is no review protocol existed. |  |
| Eligibility criteria | 6 | Studies were included if they met the following inclusion criteria: (1) the diagnosis of lung cancer was made based on a gold standard, (2) peripheral blood must have been collected for mir-21 analysis before any treatment, (3) The studies detecting mir-21 concentration in peripheral blood but not in tissue were included, (4) Studies presenting sufﬁcient data to allow construction of two-by-two tables, (5) and the literature reviewed was published in English or Chinese. | Methods |
| Information sources | 7 | Databases: Pubmed, EMBASE, Web of Knowledge (ISI), the Cochrane Library, Scopus, BioMed Central, Science Direct, China National Knowledge Infrastructure (CNKI), Wan Fang data and Technology of Chongqing (VIP). Contact with study authors to identify additional studies.  Date last searched: June 18, 2013. | Methods |
| Search | 8 | **PubMed:** (miRNA-21[Title/Abstract] or microRNA -21[Title/Abstract] or miR-21[Title/Abstract] or hsa-miR-21[Title/Abstract] ) and (lung [Title/Abstract] ) AND (cancer[Title/Abstract] OR carcinoma[Title/Abstract] OR tumor[Title/Abstract] OR neoplasm[Title/Abstract] OR cancers[Title/Abstract] or adenocarcinoma [Title/Abstract]) AND (serum[Title/Abstract] OR sera[Title/Abstract] OR serums[Title/Abstract] OR blood[Title/Abstract] OR plasma[Title/Abstract])  **Embase:**(miRNA-21:ab,ti or microRNA-21:ab,ti or miR-21:ab,ti or hsa-miR-21:ab,ti) and (lung:ab,ti) AND (cancer:ab,ti OR carcinoma:ab,ti OR tumor:ab,ti OR neoplasm:ab,ti OR cancers:ab,ti) AND (serum:ab,ti OR sera:ab,ti OR serums:ab,ti OR blood:ab,ti OR plasma:ab,ti) | Methods |
| Study selection | 9 | All publications identified by our search strategy were independently assessed by two reviewers. Disagreement about study selection was resolved by discussion and consensus. | Methods |
| Data collection process | 10 | Two reviewers independently extracted data in every study to obtain information. | Methods |
| Data items | 11 | Two-by-two table, cut-off value, sensitivity, specificity, study design, age, gender, clinical stage, first author, year of publication. | Methods |
| Risk of bias in individual studies | 12 | The publication bias of selected studies was assessed using the funnel plot, meanwhile the Begg’s test and Egger’s test were used to evaluate publication bias statistically. | Methods |
| Summary measures | 13 | The diagnostic meta-analysis was performed using a bivariate meta-analysis model to summarize the sensitivity, speciﬁcity, positive likelihood ratio (PLR), negative likelihood ratio (NLR), diagnostic odds ratio (DOR) and constructed a bivariate summary receiver operator characteristic (SROC) curve. | Methods |
| Synthesis of results | 14 | Analyses were performed using stata SE12.0 and Meta-DiSc software. Pooled sensitivity and specificity, PLR, NLR, SROC and DOR were calculated. Consistency was done by I^2^ test. | Methods |

Page 1 of 2

| **Section/topic** | **#** | **Checklist item** | **Reported on page #** |
| --- | --- | --- | --- |
| Risk of bias across studies | 15 | publication bias, selective reporting within studies | Methods |
| Additional analyses | 16 | sensitivity | Methods |
| **RESULTS** | | |  |
| Study selection | 17 | The initial search returned a total of 206 manuscripts for further detailed evaluation. For these manuscripts, 178 reviews and meta-analysis were excluded. Then 28 research articles retrieved for detailed evaluation. 7 manuscripts were excluded as imperfect information asked by Inclusion Criteria, leaving 21 available for further reviewing full text. After carefully reading the full text, Of the remaining 21 manuscripts, samples of 3 studies were not from peripheral blood, 5 studies were not diagnostic, 4 studies failed to publish value, and 2 studies were published by the same author in 2011. Thus, the meta-analysis was performed on the 7 remaining studies. | Results |
| Study characteristics | 18 | first author, year of publication, clinical stage, age, gender | Results |
| Risk of bias within studies | 19 | Funnel plots about mir-21 was used to show the publication bias in the meta-analysis. The result of the test for publication bias was not significant (*p*=0.739), the shape of funnel plots revealed a bit of asymmetry in some level due to the limited number of articles we selected(**Supplementary Fig. 1**). Then, Begg’s test and Egger’s test were performed and the P value was 0.548 and 0.058, respectively. There was no evidence that publication bias existed. However, for the limited number of the articles, whether the publication bias existed in this meta-analysis is difficult to draw a conclusion. | Results |
| Results of individual studies | 20 | Heterogeneity in sensitivity and specificity were observed among the four studies (*I*^2^=90.54% and *I*^2^=74.30%), which indicated significant heterogeneity for these included studies (Figure 2). The existence of signiﬁcant heterogeneity occurred in the 7 studies, thus the random effects model approach was selected in this study. The bivariate meta-analysis resulted in a pooled sensitivity of mir-21 for the diagnosis of lung cancer of 0.71(95%CI 0.57 – 0.82) and a pooled speciﬁcity of 0.84 (95%CI 0.76 – 0.89). | Results |
| Synthesis of results | 21 | Sensitivity: 0.71(95%CI 0.57 – 0.82, I^2^=90.54%), and speciﬁcity: 0.84 (95%CI 0.76 – 0.89 I^2^=74.30%). PLR: 4.00(95%CI: 2.4–6.67, I2=73.1%), NLR: 0.35 (95%CI: 0.23–0.52, I2= 88.0%). AUC: 0.86 (95%CI 0.83 – 0.89), and the Diagnostic Odds Ratio: 12.58 (95%CI: 5.76 – 27.48). | Results |
| Risk of bias across studies | 22 | Begg’s test and Egger’s test were performed and the P value was 0.548 and 0.058. | Results |
| Additional analysis | 23 | Sensitivity analysis determined that the meta-analysis was not influenced obviously by any individual study. | Results |
| **DISCUSSION** | | |  |
| Summary of evidence | 24 | In this meta-analysis, the pooled sensitivity and speciﬁcity were 0.71(95%CI 0.57 – 0.82) and 0.84 (95%CI 0.76 – 0.89) respectively. The results reported showed that plasma mir-21 had good accuracy in the diagnosis of lung cancer, with an area under the ROC curve of 0.86. Overall, we considered that the results of mir-21 in lung cancer were satisfactory. | Discussion |
| Limitations | 25 | Although we tried to avoid the bias in the process of meta-analysis, there were several limitations to our study. Firstly, mir-21 as a novel marker in lung cancer patients just was researched in recent years, and the diagnosis value of mir-21 was explored rarer. So study size obtained in this meta-analysis was not satisfied. Secondly, although we searched the studies published in Chinese and English, we did not search other languages or unpublished data. For these reasons, some other languages and unpublished data might not have been included in our study, which may have influences on the pooled results. Thirdly, despite of our best efforts such as by searching other related references, e-mail, and fax to all authors, we could not acquire the independent patient data (IPD) of Shen’s study for further study. | Discussion |
| Conclusions | 26 | In conclusion, despite the limitations mentioned above, the current evidence suggests that mir-21 has potential diagnostic value with moderate sensitivity and specificity in lung cancer. Larger prospective studies are needed in future, and then meta-analysis should perform more accurately. In addition, how to improve the accuracy should be considered or novel lung cancer markers with improved accuracy should be applied in future. | Discussion |
| **FUNDING** | | |  |
| Funding | 27 | No funding. |  |

*From:*  Moher D, Liberati A, Tetzlaff J, Altman DG, The PRISMA Group (2009). Preferred Reporting Items for Systematic Reviews and Meta-Analyses: The PRISMA Statement. PLoS Med 6(6): e1000097. doi:10.1371/journal.pmed1000097

For more information, visit: **www.prisma-statement.org**.

Page 2 of 2

1. Shen J, Todd NW, Zhang H, Yu L, Lingxiao X, et al. (2011) Plasma microRNAs as potential biomarkers for non-small-cell lung cancer. Laboratory Investigation 91: 579-587.
